# Comparison of clinical outcomes between carbon ion radiotherapy and X-ray radiotherapy for reirradiation in locoregional recurrence of rectal cancer

**DOI:** 10.1038/s41598-022-05809-4

**Published:** 2022-02-03

**Authors:** Seung Yeun Chung, Hirotoshi Takiyama, Jae Hyun Kang, Jee Suk Chang, Byung Soh Min, Hiroshi Tsuji, Shigeru Yamada, Woong Sub Koom

**Affiliations:** 1 Department of Radiation Oncology, Yonsei Colorectal Cancer Clinic, Yonsei Cancer Center, Yonsei University College of Medicine, 50-1 Yonsei-ro, Seodaemun-gu, Seoul, 03722 Korea; 2 Department of Radiation Oncology, Ajou University School of Medicine, Suwon, Korea; 3 Department of Radiation Oncology, QST Hospital, National Institutes for Quantum Science and Technology, 4-9-1 Anagawa, Inageku, Chiba 263-8555 Japan; 4 Department of Surgery, Yonsei Colorectal Cancer Clinic, Yonsei University College of Medicine, Seoul, Korea

**Keywords:** Radiotherapy, Rectal cancer

## Abstract

Carbon ion radiotherapy (CIRT) has garnered interest for the treatment of locoregional rectal cancer recurrence. No study has compared CIRT and X-ray radiotherapy (XRT) for reirradiation (reRT) in such cases. We analyzed and compared the clinical outcomes such as local control, overall survival, and late toxicity rate between CIRT and XRT, for treating locoregional rectal cancer recurrence. Patients with rectal cancer who received reRT to the pelvis by CIRT or XRT from March 2005 to July 2019 were included. The CIRT treatment schedule was 70.4 Gy (relative biological effectiveness) in 16 fractions. For the XRT group, the median reRT dose was 50 Gy (range 25–62.5 Gy) with a median of 25 fractions (range 3–33). Thirty-five and 31 patients received CIRT and XRT, respectively. Tumour and treatment characteristics such as recurrence location and chemotherapy treatment differed between the two groups. CIRT showed better control of local recurrence (adjusted hazard ratio [HR] 0.17; p = 0.002), better overall survival (HR 0.30; p = 0.004), and lower severe late toxicity rate (HR 0.15; p = 0.015) than XRT. CIRT was effective for treating locoregional rectal cancer recurrence, with high rates of local control and survival, and a low late severe toxicity rate.

## Introduction

Rectal cancer is one of the most common malignancies worldwide, and its treatment has progressed considerably with a multidisciplinary approach including surgery, chemotherapy, and radiotherapy (RT)^1,2^. However, despite the advancement in treatment modalities, 5–15% of the patients still develop locoregional recurrences^3^. The prognosis of such patients who cannot undergo curative R0 surgery is dismal^4^. Furthermore, uncontrolled locoregional recurrences are found to significantly affect the patients’ quality of life, leading to pelvic pain, bleeding, fistula, and urinary and faecal incontinence^5^. Thus, adequate curative treatment for locoregional pelvic recurrence is imperative.

In patients with rectal cancer with locoregional recurrence, surgery with negative margins is the preferred first-line treatment option^6^. However, many patients are ineligible for surgery due to the extent and location of tumour recurrence, or the poor general condition of the patient. A small proportion of patients are eligible for R0 resection, and other extensive resections including techniques like sacropelvic resection. But, these techniques might pose chances of functional compromise^6,7^. In such cases, RT can be considered as a feasible treatment option to improve local control and provide palliative relief^8^. However, since the majority of the recurrences are within the initial RT field, many cases will be reirradiation (reRT) cases^3^. Although the progress in RT techniques allows for higher doses to be administered at the site of the tumour and lower doses nearby, deciding on an effective radiation dose for a recurrence is challenging, considering the extent and location of recurrence at a site, especially when it is close to the bowel and bladder. Moreover, due to the RT dose accumulated from the initial administration, it is very challenging to administer reRT to achieve both an adequate radiation dose for local control and minimise toxicity, simultaneously.

Carbon ion radiotherapy (CIRT) has emerged as a subject of interest for the treatment of locoregional recurrence of rectal cancer. When compared with X-ray radiotherapy (XRT), CIRT has physical and biological advantages due to its unique characteristic of higher linear energy transfer^9,10^. Another distinct characteristic—known as the Bragg peak—allows a much reduced radiation dose to the nearby structures^11^. Thus, it can be hypothesised that CIRT might provide better local control and offer less toxicity when compared with XRT. Currently, there are a few studies from Japan and Germany reporting the efficacy of CIRT for reRT in treating locoregional recurrence of rectal cancer^12–15^. However, there are limited prospective studies, and only some retrospective studies on XRT for reRT, all showing varying median survival and toxicity rates^16^. Moreover, no study has yet compared CIRT and XRT for reRT in the treatment of locoregional recurrence of rectal cancer. Patients who receive XRT are usually more heterogenous than those who receive CIRT and this can be a limitation in comparing treatment outcomes. However, despite the possible limitations, the comparison of the two treatment methods can give important clues for choosing treatment modalities in rectal cancer patients with locoregional recurrences. Thus, the aim of this study was to analyze and compare clinical outcomes such as local control, survival, and late toxicity events in patients who received CIRT or XRT for locoregional recurrence of rectal cancer.

## Methods

### Patients and treatment

Patients who received CIRT in Japan and those who received XRT in Korea were included in this study. Medical records of patients with rectal cancer who received reRT to the pelvis with a curative intent from March 2005 to July 2019 were retrospectively reviewed. Only patients who received preoperative and postoperative RT previously as part of the initial curative treatment were included. Patients who did not receive reRT to the pelvis, those who had distant metastasis, and those who received reRT with a palliative intent were excluded. Patients with recurrence involving the anastomosis were also excluded since anastomotic recurrence was a contraindication for CIRT. Since this study included patients from two different institutions, it was approved by the Institutional Review Board of Severance hospital (IRB no. 420191320) and that of NIRS (20-013 [National Institutes for Quantum Science and Technology Certified Review Board]). Informed consent from participants was waived by the Institutional Review Board of Severance hospital and the National Institutes for Quantum Science and Technology Certified Review Board due to the retrospective nature of the study. All methods were performed in accordance with the Declaration of Helsinki.

The treatment schedule for CIRT was 70.4 Gy (relative biological effectiveness [RBE]) in 16 fractions, which is 101.38 Gy in biological effective dose with an alpha/beta ratio of 10 (BED10), without concurrent chemotherapy. CIRT was administered daily, 4 days a week from Tuesday through Friday. A median of 3 ports (range 2–5 ports) was used for treatment. The clinical target volume (CTV) was defined as gross tumour volume (GTV) with a 5-mm margin, and dose constraints of D_2cc_ for the bowel and bladder were 44 Gy (RBE) and 50 Gy (RBE) in 16 fractions, respectively. In patients who had recurrence close to the bowel or bladder (< 3 mm), Goretex soft tissue patch or biomaterials such as muscle, mesentery, or omentum were inserted as spacers between the tumour and the bowel or bladder.

For XRT, imaging studies were used for clinical diagnoses of recurrence; pathologic diagnosis was not mandatory. Planning target volume (PTV) was defined as GTV of the recurred tumour plus a 0.5–3-cm margin, depending on the physician’s preference. For cases in which the organs-at-risk (OAR) were in close vicinity, the margins were generally 0.5–1-cm, or further reduced. Dose constraints for OARs were the first priority. Both hypofractionation and conventional fractionation were used based on the physician’s preferences. Nine patients (29%) received XRT via 3D conformal RT and the rest received intensity-modulated RT or RT via CyberKnife. The median reRT dose was 50 Gy (range 25–62.5 Gy) with a median fraction number of 25 (range 3–33), which corresponds to a BED10 of 60 Gy, and 68% of the patients received concurrent chemotherapy during reRT with regimens such as capecitabine, 5-fluorouracil/leucovorin, and 5-fluorouracil/leucovorin/oxaliplatin.

### Follow-up

Patients who received CIRT were evaluated by imaging studies within 1 month of CIRT and were followed-up every 1 or 2 months for 6 months, and every 3–6 months thereafter. For the XRT group, follow-up evaluation was performed at 1 and 3 months after reRT and routinely thereafter. Acute and late toxicity events in both groups were retrospectively reviewed and graded according to the National Cancer Institute Common Terminology Criteria for Adverse Events (CTCAE) version 4.03. The primary endpoint was local failure (LF), and secondary endpoints were overall survival (OS) and severe late toxicity rate (SLTR). Local failure was defined as tumour recurrence or progression within the CTV or PTV after treatment. Severe toxicity events were defined as toxicity events of grade 3 or higher.

### Statistical analysis

Clinical factors between the two groups were analyzed by χ^2^ and Fisher’s exact tests. The cumulative probabilities of LF, OS, and SLTR were calculated using the Kaplan–Meier method and compared using the log rank test. All LF, OS, and SLTR were defined as time from reRT until the corresponding events or the date of last follow-up. Cox regression analysis was used for univariate and multivariate analyses. For multivariate analysis, the backwards elimination method including all variables was used. All analyses were performed using the SPSS version 23.0 (IBM Inc., Armonk, NY, USA).

## Results

Totally, 35 patients who received CIRT (CIRT group) and 31 patients who received XRT (XRT group) were analyzed. The characteristics of patients and tumours as well as details regarding treatments are shown in Table 1. Patient characteristics such as sex, age, and initial tumour stage showed no statistically significant differences between the groups. In the CIRT group, approximately half of the patients experienced recurrence in the presacral area (51%), whereas the major location of the recurred lesion was non-presacral, regional, or nodal (71%) in the XRT group. The median size of the recurred tumour was 2.5 cm and 3.0 cm in the CIRT and XRT groups, respectively. A higher number of patients in the XRT group (74%) received chemotherapy before or after reRT compared with those in the CIRT group (40%). No patients in the CIRT group received concurrent chemotherapy, while 68% of the patients in the XRT group received concurrent chemotherapy during reRT. All patients in the CIRT group received CIRT without any surgery before or after RT, while 30% of the patients in the XRT group (n = 11) underwent surgery before or after RT.

**Table 1:** Patient, tumour, and treatment characteristics, n = 66.

	CIRT		XRT		p
	N	%	N	%	
**Sex**					0.732
Male	20	57	19	61	
Female	15	43	12	39	
**Age**					0.926
Median (range)	62 (37–76)		60 (35–87)		
**Initial**					
Initial pathology					0.470*
Adenocarcinoma	35	100	30	97	
Mucinous	0	0	1	3	
Initial grade					0.026*
G1	11	31	4	13	
G2	13	37	23	74	
G3	4	11	1	3	
Unknown	7	20	3	10	
Initial tumour size					0.856
Median (mm, range)	30 (13–70)		31 (0–70)		
Initial pathologic T stage					0.965*
T0	1	3	1	3	
T1	2	7	2	6	
T2	3	10	2	6	
T3	21	70	24	77	
T4	3	10	2	6	
Initial pathologic N					0.861*
Positive	18	51	18	58	
Negative	12	34	9	29	
Unknown	5	14	4	13	
Initial pathologic stage					0.123
Stage 0	0	0	1	3	
Stage I	4	13	0	0	
Stage II	8	27	12	39	
Stage III	18	60	18	58	
Initial RT type					0.429
Preoperative RT	24	71	19	61	
Postoperative RT	10	29	12	39	
Previous RT total dose (cGy)					0.003
Median (range)	5000 (2000–6600)		5040 (4500–6000)		
**Recurrence**					
Recur location					0.082
Non-presacral, regional, nodal	17	49	22	71	
Presacral	18	51	9	29	
Recurred tumour size					0.450
Median (mm, range)	25 (15–80)		30 (10–70)		
Recur : lymph node					0.615
Negative	24	69	23	74	
Positive	11	31	8	26	
Pre- or post-RT chemotherapy					0.005
No	21	60	8	26	
Yes	14	40	23	74	
Concurrent chemotherapy					< 0.001
No	35	100	10	32	
Yes	0	0	21	68	
ReRT total dose (cGy)					< 0.001
Median (range)	7040 (7040–7040)		5000 (2500–6250)		< 0.001
ReRT fraction					
Median (range)	16 (16–16)		25 (3–33)		
Surgery					0.001
After reRT	0	0	7	23	
Before reRT	0	0	4	13	
ReRT only	35	100	20	65	

carbon ion radiotherapy, *XRT* X-ray radiotherapy, *RT* radiotherapy.

*Fisher’s exact test.

**Figure 1:**
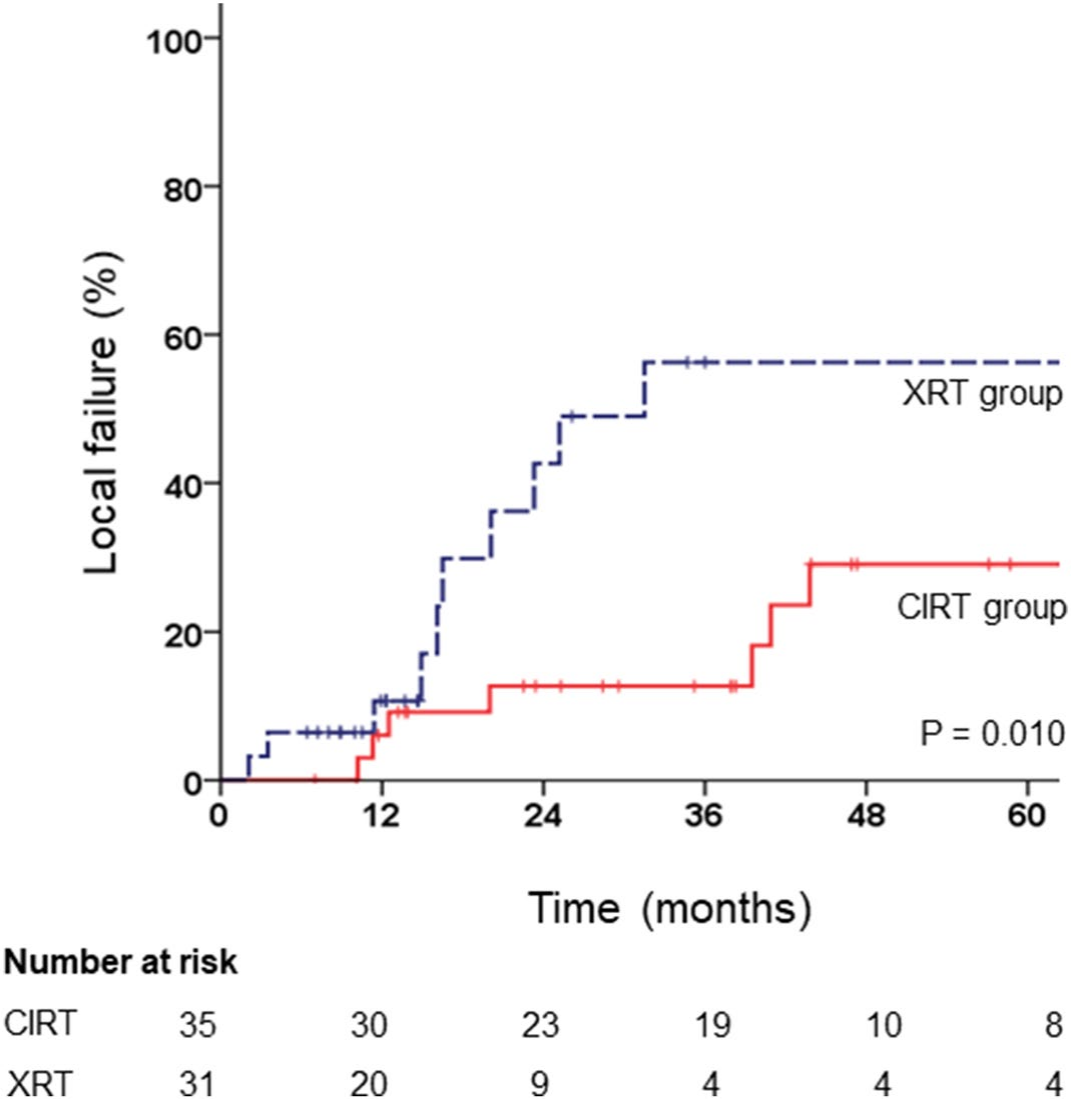
Kaplan–Meier estimates of local failure according to treatment groups.

The median follow-up period was 45.7 months (range 7.0–148.4 months) and 22.8 months (range 7.2–148.4 months) in the CIRT and XRT groups, respectively (p = 0.966). One-year LF rates were 6.1% and 10.7% and 3-year LF rates were 12.7% and 56.3% in the CIRT and XRT groups, respectively (Fig. 1, p = 0.010). A total of 7 patients experienced LF in the CIRT group and 6 patients received a second CIRT with the same dose-fractionation schedule. In the XRT group, a total of 11 patients experienced LF. When other factors were adjusted, receiving CIRT compared with XRT was a statistically significant favourable factor for LF (Table 2, adjusted hazard ratio [HR] 0.17; 95% confidence interval [CI] 0.05–0.51; p = 0.002). One-year OS rates were 97.0% and 88.9% and 3-year OS rates were 86.4% and 54.5%, in the CIRT and XRT groups, respectively. (Supplementary Fig. 1, p = 0.005). While median survival in the CIRT group was not achieved, the median survival was 36.9 months in the XRT group. Multivariate analysis showed that an increase in tumour size per millimetre was a statistically significant unfavourable factor (HR 1.04; CI 1.02–1.06; p < 0.001), while CIRT compared with XRT was a significant favourable factor (HR 0.30; CI 0.13–0.68; p = 0.004) for OS (Supplementary Table 1).

**Table 2:** Univariate and multivariate analysis for factors associated with the primary end point of local failure (n = 66).

Factor	Uni	Multivariate	p
	p	HR(95% CI)	
Sex (female vs. male)	0.643		
Age (years)	0.783		
Recur location (presacral vs. non-presacral)	0.089	4.30 (1.47–12.59)	0.008
rN stage (rN + vs. rN0)	0.246		
Pre or post-RT chemotherapy (yes vs. no)	0.208		
Pre or post-RT surgery (yes vs. no)	0.473		
Recurred tumour size (mm)	0.110	1.03 (1.00–1.05)	0.054
Treatment (carbon ion therapy vs. X-ray therapy)	0.015	0.17 (0.05–0.51)	0.002

*RT* radiotherapy, *N* nodal.

Acute toxicity was similar between the groups. Acute gastrointestinal (GI) toxicity of grade 2 or higher was seen in 1 (3%) and 4 patients (13%) in the CIRT and XRT groups, respectively. Acute genitourinary (GU) toxicity of grade 2 or higher was seen in 3 (9%) and 2 (6%) patients in the CIRT and XRT groups, respectively. As shown in Table 3, severe late GI toxicity events were seen in 2 patients in the CIRT group, with a median time to event of 12 months (range 5.8–18.2 months), compared with 6 patients in the XRT group with a median time to event of 8.6 months (range 4.3–30.0 months). No events of severe late GU toxicity were observed in the CIRT group, while they occurred in 4 patients in the XRT group, with a median time to event of 12.2 months (range 6.9–14.7 months).

**Table 3:** Prevalence of severe late toxicity events amongst patients receiving CIRT and XRT, n = 66.

	CIRT		XRT	
	n	%	n	%
**Severe late GI toxicity**				
No	33	94	25	81
Yes	2	6	6	19
Time to event (months)	12.0 (5.8–18.2)		8.6 (4.3–30.0)	
**Severe late GU toxicity**				
No	35	100	27	87
Yes	0	0	4	13
Time to event (months)			12.2 (6.9–14.7)	

*CIRT* carbon ion radiotherapy, *XRT* X-ray radiotherapy, *GI* gastrointestinal, *GU* genitourinary.

When seen in detail, the location of the recurred tumour in patients who experienced severe late GI toxicity was presacral (n = 2, 6%) in the CIRT group while that in the XRT group was both presacral and non-presacral (Supplementary Table 2).

**Figure 2:**
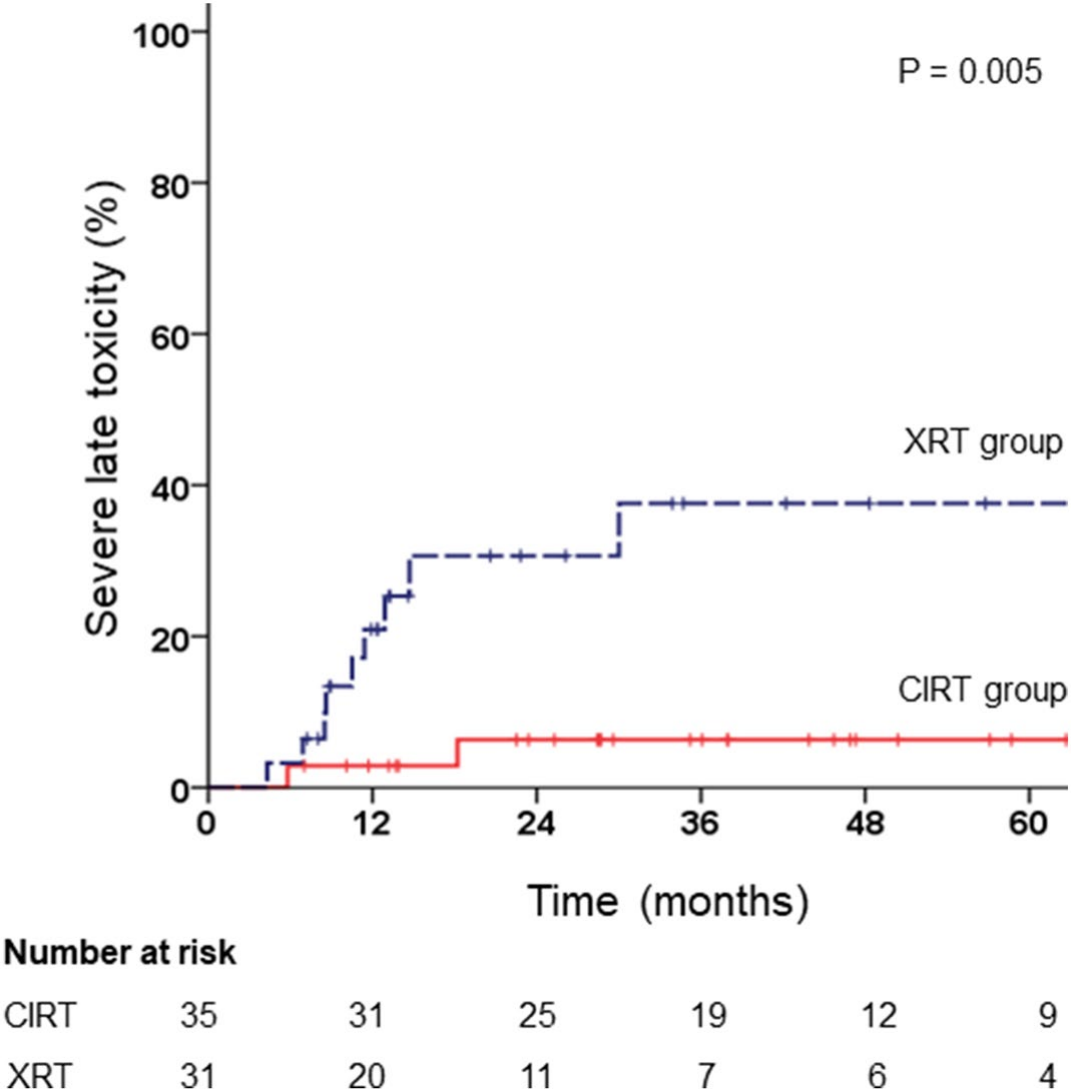
Kaplan–Meier estimates of severe late toxicity rate according to treatment groups.

In the CIRT group, 1 out of 2 patients who experienced severe late GI toxicities received bevacizumab after CIRT. In the XRT group, 3 out of 6 patients who experienced severe late GI toxicities received bevacizumab prior to XRT. In addition, out of 6 patients who experienced severe late GI toxicity in the XRT group, 5 patients (83%) received chemotherapy before or after reRT. All 4 patients who experienced severe late GU toxicity in the XRT group received chemotherapy before or after reRT. Patients in the XRT group who experienced severe late GI or GU toxicity showed a shorter median period from initial RT to reRT. Skin toxicity was seen in 4 patients (11%) in the CIRT group, in the form of skin ulcers and skin tumour fistula, and in 2 patients (6%) in the XRT group, in the form of skin reaction and postoperative skin dehiscence. For analysis of SLTR, severe late GI and GU toxicity events were included. The 1-year and 3-year SLTRs were 2.9% and 6.3%, respectively, in the CIRT group, and 20.9% and 37.6% in the XRT group, respectively (Fig. 2, p = 0.005).

After adjusting for other factors, CIRT compared with XRT was a significant favourable factor for less severe late toxicity (HR 0.15; CI 0.30–0.69; p = 0.015) (Table 4).

**Table 4:** Univariate and multivariate analysis for factors associated with severe late toxicity.

Factor	Uni	Multivariate	p
	p	HR(95% CI)	
Sex (female vs. male)	0.362		
Age (years)	0.234		
Recur location (presacral vs. non-presacral)	0.392		
rN stage (rN + vs. rN0)	0.817		
Pre or post-RT chemotherapy (yes vs. no)	0.080		
Pre or post-RT surgery (yes vs. no)	0.491		
Recurred tumour size (mm)	0.557		
Treatment (carbon ion therapy vs. X-ray therapy)	0.015	0.15 (0.3–0.69)	0.015

*RT* radiotherapy, *N* nodal.

## Discussion

Treatment of locoregional recurrence of rectal cancer in the pelvis remains a challenge for clinicians. Preoperative RT is frequently considered as the initial treatment strategy; thus, the majority of the locoregional recurrences are cases that require reRT^3^. In this study, we showed the efficacy of CIRT as a treatment modality for locoregional recurrence of rectal cancer. CIRT was found to be preferable for local control, preventing severe late toxicity, and providing better OS when compared with XRT.

Previously, a limited number of prospective trials and some retrospective studies about reRT using XRT in locoregional recurrence of rectal cancer have been reported^8,17^. The rates of local control, survival, and late toxicity varied widely in these studies given the heterogeneity of the patients. In these previous studies, the overall rates of local control generally ranged from 25 to 70%. Patients who did not undergo any surgery had a median survival of 10–17 months, while those who underwent resection had a median survival of 22–60 months^8,17^. In our study, the XRT group included a mix of patients who, whether they underwent resection of the recurred tumour or not, had a median survival of 36.9 months. One-year and 3-year rates of LF were 10.7% and 56.3%, respectively, which are within the range reported by previous studies^8,17^. Late toxicity has been inadequately reported in previous studies, and the rates of severe late toxicity vary widely, ranging from 12 to 39%^16–18^. Late toxicity events usually include small bowel obstruction, urinary obstruction, hydronephrosis, and fistula formation. In patients receiving reRT, it is difficult to clearly determine if the late toxicity events are caused by the treatment or by the recurrence of the tumour; hence, assessment of late severe toxicity is challenging. In this study, the rate of severe late toxicity events in the XRT group was similar to that reported in previous studies.

Considering that clinical outcomes of the XRT group in this study were in line with those reported in previous studies, the clinical outcomes of the CIRT group are very encouraging. Despite differences in some recurred tumour or treatment characteristics, patients in the CIRT group showed excellent results in terms of local control, OS, and fewer late toxicity events. CIRT physical properties of dose deposition allowed the delivery of a higher dose to the target volume. When the median prescribed doses were converted to BED10 for comparison, the median BED10 for the CIRT group was 101.4 Gy, whereas it was 60 Gy in the XRT group. This might have led to better local control in the CIRT group compared with that in the XRT group, since higher doses could be related to higher local control^19^. Moreover, previous studies reported that rectal adenocarcinoma and its recurrence show significantly increased hypoxia, which leads to radio-resistance^20,21^. In such cases, the unique characteristic of CIRT, which is a high linear energy transfer, provides a superior biological effect by causing direct double strand breaks^9^. Consequently, effective local control of the recurred tumour by CIRT may have led to an increased OS by reducing distant metastases and overall progression^22^. It is notable that the patients in the CIRT group showed excellent local control and OS even without resection of the tumour, as R0 resection is generally considered as the most important factor for survival. Since only a limited number of patients can undergo curative resection, CIRT may act as an effective strategy for treating locoregional recurrence of rectal cancer. In addition, most patients with LF after CIRT were treated with reRT using CIRT again.

CIRT showed low rates of severe late toxicity. Another unique characteristic of CIRT is the Bragg peak, which allows lesser radiation to the organs at risk, thereby lowering the late toxicity rates^10^. When compared with the CIRT group, a higher proportion of patients in the XRT group received pre- or post-RT chemotherapy. Chemotherapy can also be associated with higher rates of toxicity^23^. Furthermore, more aggressive measures to protect OARs, such as the spacer insertion, were taken in the CIRT group. Thus, while the low rates of late toxicity in the CIRT group can be accepted, direct comparison of the severe late toxicity rates between the CIRT and XRT groups has its limitations.

Due to the advantage of CIRT in the locoregional recurrence of rectal cancer, favorable results from a prospective observational study (GUNMA 0801) has been reported and further studies on CIRT in the treatment of recurrent rectal cancer are ongoing, including the HIMAT1351 and PANDORA01 trials^15,24,25^. Nevertheless, despite the clinical advantages, the cost of CIRT can be a concern for the patients. In Japan, a cost-effectiveness study showed that CIRT can be potentially cost-effective compared with the multimodality treatment^26^. Further studies about CIRT in locoregional recurrence of rectal cancer are anticipated.

The limitations of this study are mainly due to its retrospective nature. Two groups from two different institutions in two different countries were included in this study, thus increasing the heterogeneity in patient, tumour, and treatment characteristics. A propensity score matching could have reduced the heterogeneity; however, this was not possible due to the less number of patients. We focused on comparing clinical results in patients treated with CIRT and XRT with curative intent. Moreover, late toxicity events were seen retrospectively, which might have affected the results. However, this study is unique in that it is the only study comparing the efficacy of CIRT and XRT in locoregional recurrence of rectal cancer.

## Conclusion

In conclusion, reRT with CIRT is an effective treatment modality for locoregional recurrence of rectal cancer, showing high rates of local control and survival and low rates of late toxicity, compared with XRT. CIRT may be an encouraging strategy in these patients.

## Supplementary Information

Supplementary Information 1.

Supplementary Information 2.

## Data Availability

The datasets generated during and/or analyzed during the current study are available from the corresponding author on reasonable request.
