# Supplementary figures and images for: Comparison of clinical outcomes between carbon ion radiotherapy and X-ray radiotherapy for reirradiation in locoregional recurrence of rectal cancer
Source: Sci Rep. 2022 Feb 3;12:1845. doi: 10.1038/s41598-022-05809-4 (PMC8813922; doi:10.1038/s41598-022-05809-4)

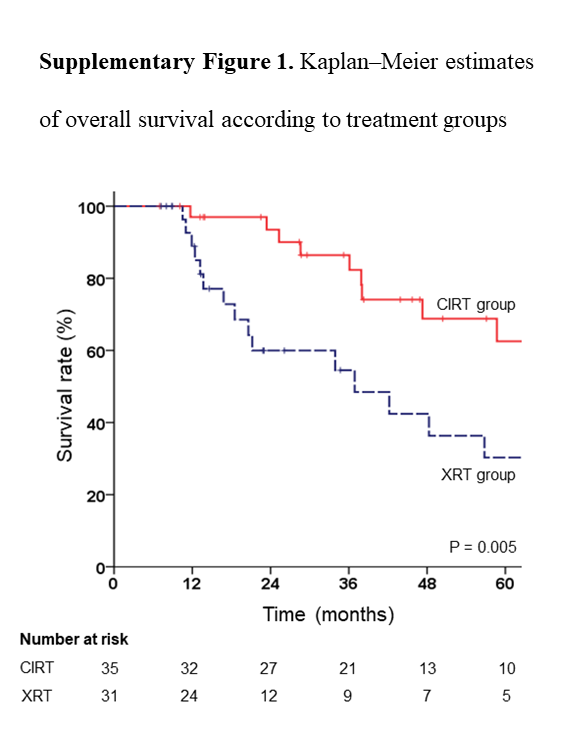

Supplement: Supplementary file 1 — Supplementary Information 1. [file 41598_2022_5809_MOESM1_ESM.tif]
