# Supplementary material for: Comparison of clinical outcomes between carbon ion radiotherapy and X-ray radiotherapy for reirradiation in locoregional recurrence of rectal cancer
Source: Sci Rep. 2022 Feb 3;12:1845. doi: 10.1038/s41598-022-05809-4 (PMC8813922; doi:10.1038/s41598-022-05809-4)
Supplement: Supplementary file 2 — Supplementary Information 2. [file 41598_2022_5809_MOESM2_ESM.docx]

| Supplementary Table 1. Univariate and multivariate analysis for overall survival | | | | |  |
| --- | --- | --- | --- | --- | --- |
|  |  |  |  |  |  |
|  |  | Uni |  | Multivariate |  |
| Factor |  | p |  | HR(95% CI) | p |
| Sex (female vs. male) |  | 0.611 |  |  |  |
| Age (years) |  | 0.747 |  |  |  |
| Recur location (presacral vs. non-presacral) |  | 0.736 |  |  |  |
| rN stage (rN+ vs. rN0) |  | 0.636 |  |  |  |
| Pre or post-RT chemotherapy (yes vs. no) |  | 0.166 |  |  |  |
| Pre or post-RT surgery (yes vs. no) |  | 0.599 |  |  |  |
| Recurred tumor size (mm) |  | <0.001 |  | 1.04 (1.02–1.06) | <0.001 |
| Treatment (Carbon ion therapy vs. X-ray therapy) |  | 0.007 |  | 0.30 (0.13–0.68) | 0.004 |

| Supplementary Table 2. Severe late toxicity events according to tumor and treatment characteristics | | | | | | | | |  |  |  |  |  |  |  |  |  |  |  |
| --- | --- | --- | --- | --- | --- | --- | --- | --- | --- | --- | --- | --- | --- | --- | --- | --- | --- | --- | --- |
|  |  |  |  |  |  |  |  |  |  |  |  |  |  |  |  |  |  |  |  |
|  | Carbon |  |  |  |  |  |  |  |  |  | X-ray |  |  |  |  |  |  |  |  |
|  | Severe late GI toxicity | | |  |  | Severe late GU toxicity | | |  |  | Severe late GI toxicity | | |  |  | Severe late GU toxicity | | |  |
|  | No |  | Yes |  |  | No |  | Yes |  |  | No |  | Yes |  |  | No |  | Yes |  |
|  | n | % | n | % |  | n | % | n | % |  | n | % | n | % |  | n | % | n | % |
| Location |  |  |  |  |  |  |  |  |  |  |  |  |  |  |  |  |  |  |  |
| Non-presacral, regional, nodal | 17 | 52% | 0 | 0% |  | 17 | 49% | 0 | 0% |  | 19 | 76% | 3 | 50% |  | 20 | 74% | 2 | 50% |
| Presacral | 16 | 48% | 2 | 100% |  | 18 | 51% | 0 | 0% |  | 6 | 24% | 3 | 50% |  | 7 | 26% | 2 | 50% |
| Pre or post-RT chemotherapy |  |  |  |  |  |  |  |  |  |  |  |  |  |  |  |  |  |  |  |
| No | 20 | 61% | 1 | 50% |  | 21 | 60% | 0 | 0% |  | 7 | 28% | 1 | 17% |  | 8 | 30% | 0 | 0% |
| Yes | 13 | 39% | 1 | 50% |  | 14 | 40% | 0 | 0% |  | 18 | 72% | 5 | 83% |  | 19 | 70% | 4 | 100% |
| Intital RT to reRT interval (mo) |  |  |  |  |  |  |  |  |  |  |  |  |  |  |  |  |  |  |  |
| Median (range) | 37.7 (13.5-157.7) | | 48.1 (20.9-75.3) | |  | 37.7 (13.5-157.7) | |  |  |  | 30.2 (6.6-138.9) | | 20.9 (14.1-73.2) | |  | 29.2 (6.6-138.9) | | 23.8 (21.9-44.9) | |
